# How will telehealth change primary care in Australia?

**DOI:** 10.3399/BJGPO.2021.0186

**Published:** 2022-01-26

**Authors:** Simon Mark Willcock, John A Cartmill, Tim Tse, Sarah J White, Amy Nguyen, Peter Roger

**Affiliations:** 1 Department of Primary Care, Faculty of Medicine, Health and Human Sciences, Macquarie University, Macquarie Park, NSW, Australia; 2 Macquarie Medical School, Faculty of Medicine, Health and Human Sciences, Macquarie University, Macquarie Park, NSW, Australia; 3 St Vincent’s Clinical School, UNSW Medicine, UNSW Sydney, Sydney, NSW, Australia; 4 Centre for Health Systems and Safety Research, Australian Institute of Health Innovation, Macquarie University, Macquarie Park, NSW, Australia; 5 Department of Linguistics, Faculty of Medicine, Health and Human Sciences, Macquarie University, Macquarie Park, NSW, Australia

**Keywords:** Telehealth, Primary Care, Communication

As a consequence of the COVID-19 pandemic, health systems have seen sudden and significant increases in the use of telehealth services.^1^ The Australian population has shared in this experience, with studies showing a rapid uptake of telehealth services.^2^ A high level of satisfaction with telehealth consultations in Australia has been reported, but some responders report reduced satisfaction compared to traditional face-to-face consultations.^3^

Australian citizens have access to a range of medical services through Medicare, the national health insurance system. The Commonwealth Health Department is responsible for funding those components of primary care services in Australia that are included within Medicare, with a defined fee for each service. The Medicare schedule of fees identifies the payment available for each item and defines the criteria for claiming each item.

The Commonwealth Department of Health website acknowledges the role of videoconferencing, but still contextualises its value as an access tool for geographically remote populations.^4^

Australian GPs have been requesting new Medicare item numbers for telehealth service provision since the technology to deliver these services became widely available. These remote-access technologies have pervaded most aspects of our everyday lives over the past 20 years, including shopping, banking, insurance, and even finding a partner. It is predictable that service providers and consumers are looking for opportunities to use these technologies to deliver some components of health services.

Despite persistent lobbying from practitioners, the Commonwealth has consistently refused to extend Medicare subsidies for telehealth services except when ‘remoteness‘ was quantifiable in geographic terms. The rationale was that, while physical distance required innovative service delivery solutions, free access to telehealth services would potentially result in overuse or frank abuse of the insurance system and potentially lower the standard of care provided.

The advent of the COVID-19 pandemic, with its attendant lockdown, isolation, and distancing requirements, resulted in a rapid and laudable review of this position, with the expedited approval of a broad range of specialist and primary care services that could be delivered in non-contact settings via technology and billed under Medicare. In summary, the government, the community, and health service providers were enrolled in huge national experiment, one where the normal development processes for research hypotheses and interventions have been ‘fast-tracked’.

After 18 months of access to telehealth services, this service format has now become established and indeed expected within the Australian community. It seems unlikely (and politically unwise) that access to these services will be completely withdrawn in the future.

It is therefore timely to consider what effects this abrupt disruption to our primary care service model has had in terms of a range of outcomes, included those articulated by the Bodenheimer’s ’quadruple aim’ of health care, summarised as good health outcomes, alignment with best practice evidence, financial sustainability, and consumer and provider satisfaction/acceptability.^5^

The World Health Organization (WHO) and United Nations Children’s Fund (UNICEF) jointly define primary care as ’*a whole-of-society approach to health that aims at ensuring the highest possible level of health and well-being and their equitable distribution by focusing on people’s needs and as early as possible along the continuum from health promotion and disease prevention to treatment, rehabilitation and palliative care, and as close as feasible to people’s everyday environment’*.^6^ An unwieldy statement that can be summarised as ’people need a healthy living environment and timely and equitable access to the health services that they require’.

When we describe the fundamental principles of primary care in Australia, we emphasise that ours is not a disease-focused model of care, but rather a person-centred model that embraces constructs such as continuity of care with service providers, team-based care, and integrated care for those with chronic and complex comorbidities.

As we adopt telehealth as a routine part of our consultations, we therefore need to ask some important questions. Does telehealth improve patient access to care? Does telehealth improve both immediate and longer-term patient outcomes (or at least maintain the outcomes achieved through face-to-face services)? Does telehealth maintain, enhance, or detract from patient and provider satisfaction in primary care service provision? Is telehealth a cost-effective model of care, and of course, what services cannot be provided satisfactorily through telehealth?

The answer to many of these questions will be influenced by the quality of the interaction between the service provider and the service consumer. Services that require detailed physical examination or a procedure to be undertaken cannot currently be delivered remotely, acknowledging that data collection from wearable devices is already facilitating consultations, and more complex robotic procedures may be possible in the future.

While considering the opportunities and value of telehealth we need to examine closely the limitations and what might be lost through its rapid and extensive adoption. The phenomenon of ’revenge effects of technology’^7^ behoves the profession to anticipate and monitor for these downsides and prepare to mitigate them.

Medicine in some form is as old as civilisation and the construct of ’care for the infirm’ may have been an enabler for civilisation itself. Our medical system is built on layers of convention, expectation, and interpretation, many of which may be so subtle as to be subliminal. It is possible that these subtle levels of meaning may not make it through the limited bandwidth of a telehealth consultation that may be further downgraded by whatever distractions the technology enables (at either end).

What situational factors will influence the successful use of telehealth? Arguably those without the means, facility, or the access to a computer and the private place to use it will have a quite different experience to the intimate, door-closed consultation where they might discuss their deepest fears. A hearing-impaired person has significant additional technology requirements. How might a person with a controlling or violent partner use telehealth?

The answer to some of these questions requires a better understanding of how telehealth influences the various subtle communication factors that enhance both patient satisfaction and patient outcomes. This requires the development of ‘telehealth literacy’ in both consumers and providers. Fortunately, homo sapiens is an adaptive species and has already shown itself to have a remarkable ability to adapt to and adopt new ways of sharing the sort of trust and emotional intimacy that effective health consultations require.
